# Human seasonal coronavirus neutralization and COVID‐19 severity

**DOI:** 10.1002/jmv.27937

**Published:** 2022-07-18

**Authors:** David A. Wells, Diego Cantoni, Martin Mayora‐Neto, Cecilia Di Genova, Alexander Sampson, Matteo Ferrari, George Carnell, Angalee Nadesalingam, Peter Smith, Andrew Chan, Gianmarco Raddi, Javier Castillo‐Olivares, Helen Baxendale, Nigel Temperton, Jonathan L. Heeney

**Affiliations:** ^1^ Department of Veterinary Medicine, Lab of Viral Zoonotics University of Cambridge Cambridge UK; ^2^ DIOSynVax University of Cambridge Cambridge UK; ^3^ Viral Pseudotype Unit, Medway School of Pharmacy University of Kent Medway UK; ^4^ Royal Papworth Hospital NHS Foundation Trust Cambridge UK

**Keywords:** endemic infection, epidemiology, neutralization, immune responses, SARS coronavirus, virus classification

## Abstract

The virus severe acute respiratory syndrome coronavirus 2 (SARS‐CoV‐2), responsible for the global coronavirus disease‐2019 (COVID‐19) pandemic, spread rapidly around the world causing high morbidity and mortality. However, there are four known, endemic seasonal coronaviruses in humans (HCoVs), and whether antibodies for these HCoVs play a role in severity of COVID‐19 disease has generated a lot of interest. Of these seasonal viruses NL63 is of particular interest as it uses the same cell entry receptor as SARS‐CoV‐2. We use functional, neutralizing assays to investigate cross‐reactive antibodies and their relationship with COVID‐19 severity. We analyzed the neutralization of SARS‐CoV‐2, NL63, HKU1, and 229E in 38 COVID‐19 patients and 62 healthcare workers, and a further 182 samples to specifically study the relationship between SARS‐CoV‐2 and NL63. We found that although HCoV neutralization was very common there was little evidence that these antibodies neutralized SARS‐CoV‐2. Despite no evidence in cross‐neutralization, levels of NL63 neutralizing antibodies become elevated after exposure to SARS‐CoV‐2 through infection or following vaccination.

## 1 INTRODUCTION

The endemic human coronaviruses (HCoVs), sometimes referred to as seasonal coronaviruses, are a group of four viruses that include the alphacoronaviruses 229E, NL63, and two betacoronaviruses HKU1, OC43. Frequently infecting humans through life and generally causing symptoms of a common cold,^1^ they are considered to be low morbidity pathogens. On rare occasions, however, infection in individuals with serious underlying diseases can lead to severe pneumonia and death.^2^, ^3^ The HCoVs are known to have a wide tropism, however, there are selective binding profiles to certain sugars and proteins. HKU‐1 and OC43 bind to sialic acid,^4^ 229E binds to human aminopeptidase N^5^ and NL63 binds to the angiotensin‐converting enzyme 2 (ACE2) receptor.^6^ The severe acute respiratory syndrome coronavirus 2 (SARS‐CoV‐2) has been rapidly transmitted and spread globally, causing over 200 million cases and more than 4 million deaths as of September 2021. Previous studies have demonstrated pre‐existing immune responses to SARS‐CoV‐2 in people not exposed to the virus. This has been reported both for antibodies,^7^, ^8^, ^9^ and T cell responses.^10^, ^11^ Though still under debate^12^, ^13^ this pre‐existing immunity to a novel virus has largely been attributed to the four widely circulating HCoVs.

There has been great interest in the potential role of common cold coronaviruses in modulating the severity of COVID‐19 disease. This is partly due to the fact that NL63 also uses the same ACE2 as its cellular receptor; therefore, it was questioned whether antibodies raised against NL63 would also bind to SARS‐CoV‐2. A recent report investigated whether seasonal HCoVs could protect against SARS‐CoV‐2 infection.^14^ Anderson et al. used antibody‐binding assays (ELISAs) to quantify antibodies against the HCoVs. However, these binding assays do not discriminate between neutralizing and non‐neutralizing antibodies. Pseudotype viruses (PVs) can be used to quantify neutralizing antibodies and have been shown to correlate with live‐virus neutralization.^15^, ^16^, ^17^

Herein, we used PVs bearing the Spike protein of SARS‐CoV‐2 and the seasonal HCoVs: NL63, HKU1, and 229E to investigate the relationships between common cold coronavirus immune responses and COVID‐19 severity in healthcare workers and COVID‐19 patients. We found that HCoV neutralization did not correlate with SARS‐CoV‐2 neutralization. This builds on previous work showing that HCoV binding does not protect against COVID‐19^14^; however, we also show that HCoV binding and HCoV neutralization are not strongly correlated. This finding highlights the importance of functional antibody assays, in addition to binding, when characterizing antibody responses. Despite a lack of cross‐neutralization, we found that NL63 neutralization is boosted by SARS‐CoV‐2 vaccination and elevated after moderate to mild COVID‐19 disease.

## 2 METHODS

### 2.1 Subject recruitment and plasma collection

Healthcare workers (HCWs) and COVID‐19 patients were recruited from Royal Papworth Hospital, Cambridge, UK in the spring 2020. HCW were recruited through staff email over the course of 2 months (April 20, 2020–June 10, 2020) as part of a prospective study to establish seroprevalence and immune correlates of protective immunity to SARS‐CoV‐2. Following informed consent, staff were invited to complete a questionnaire to clarify whether they had swab polymerase chain reaction confirmed SARS‐CoV‐2 infection (routine swabbing was not available at that time and there was limited access to swabbing when symptomatic) and whether they had experienced symptoms that they felt may have been consistent with COVID‐19 since January 2020. Symptom severity was classified according to WHO severity classification into asymptomatic, mild, moderate, and severe disease.^18^ The study was approved by Research Ethics Committee Wales, IRAS 96194 12/WA/0148. Amendment 5. All participants provided written and informed consent before being enrolled in this study.

Plasma was taken from HCWs and convalescent COVID‐19 patients 3–5 months after recruitment by collecting venous blood in lithium heparin tubes (S‐Monovette) and centrifuged at 2300G. Without disturbing the buffy coat, the plasma was transferred into 1.5 ml cryovials and heat‐inactivated at 56°C for 30 min, aliquoted and stored at −80°C before use. We analyzed SARS‐CoV‐2 neutralization and HCoV neutralization for 38 COVID‐19 patients, 23 seropositive HCWs, and 39 seronegative HCWs. These samples are described in more detail in Castillo‐Olivares et al.^19^ We had a particular interest in NL63 because along with SARS‐CoV2 it uses the ACE2 receptor to enter cells. Because of our interest in NL63, we analyzed a further set of samples for SARS‐CoV‐2 and NL63 neutralization: 35 seropositive HCWs, 140 seronegative HCWs, and 7 COVID‐19 patients (six were seropositive). We also collected follow‐up samples from 21 of our HCWs 1 month after they received their first SARS‐CoV‐2 vaccination dose, approximately 9–12 months after they were first recruited to our study.

### 2.2 Classifying sample serostatus

Samples' serostatus was determined according to SARS‐CoV‐2 IgG binding status. This was determined by neutralization (SARS‐CoV‐2 pMN IC50), and/or IgG binding to SARS‐CoV‐2 Spike, Nucleocapsid, and Spike receptor‐binding domain (RBD) by a UKAS‐accredited Luminex assay as described in.^19^ These two methods of classification showed good agreement but as neither assay was performed on all samples a positive result on either assay classified the sample as seropositive. The seropositive cutoff for pMN was the 95% upper confidence interval of pre‐pandemic samples in previous work.^19^ The classification based on IgG binding is described in Baxendale et al.,^20^ but in brief, a linear support vector machine was trained to distinguish a set of pre‐pandemic sera from COVID‐19 patient sera. This classification method considers the three antigens jointly so there is no single cut‐off to report.

### 2.3 Tissue culture

Human Embryonic Kidney cells (HEK293T/17) cells and Huh‐7‐based hepatoma cells were maintained using Dulbecco's Modified Eagle Medium (DMEM) supplemented with 10% fetal bovine serum (FBS) and 1% penicillin/streptomycin (P/S). Chinese Hamster Ovary (CHO) cells were maintained in Ham's F‐12 medium supplemented with 10% FBS and 1% P/S. All cells were incubated at 37°C and 5% CO_2_. Cells were routinely passaged three times a week to prevent overconfluency.

### 2.4 Pseudotype virus generation

PV generation was carried out as previously described.^21^ Plasmids bearing the Spike of either SARS‐CoV‐2 (Ancestral strain, YP_009724390), NL63 (YP_003767), HKU1 (YP_173238), or 229E (NP_073551) in the vector pcDNA3.1+, were mixed with the plasmids p8.91 lentiviral Gag‐pol^22^ and pCSFLW luciferase reporter gene^23^ in Opti‐MEM solution. Plasmids were mixed and incubated for 15 min with FuGENE‐HD transfection reagent, followed by dropwise addition onto HEK293T cells in T‐75 flasks. For HKU1 PVs, 1.5U of exogenous neuraminidase (Sigma) was added in 10 ml of replenished DMEM 18–24 h posttransfection. Cells were incubated for 48 h before removal and filtering of the supernatant culture media through 0.45 µM cellulose acetate membranes. Aliquots of filtered supernatant were stored at −80°C. Pseudotypes were titrated in white flat‐bottomed 96 well plates by serially diluting twofold into DMEM for PVs bearing the SARS‐CoV‐2 and NL63 spike, or Ham's F‐12 media for PVs bearing the spike of HKU1. Target cells for SARS‐CoV‐2 and NL63 PVs were HEK293T cells pre‐transfected with ACE‐2 and TMPRSS‐2,^24^ and CHO cells were used as target cells for HKU1 PVs. Plates were incubated at 37°C and 5% CO_2_ for 48 h before lysis using Bright‐Glo and assaying luciferase reporter gene activity in relative light units (RLU) using a luminometer. PV titers were reported in RLU/ml.

### 2.5 Pseudotype virus neutralization (pMN) assays

pMN assays were carried out as previously described.^21^ Briefly, plasma was mixed with either DMEM or Ham's F‐12 depending on the PV, at an initial 1:40 dilution and serially diluted 2‐fold in white flat‐bottomed 96‐well plates to a final 1:5,120 dilution. PVs were then added to the wells at an input of 5 × 10^5^ RLU/ml and plates were incubated at 37°C and 5% CO_2_ for 1 h. Pre‐transfected HEK293T target cells were seeded at 1×10^4^ cells per well in plates containing either SARS‐CoV‐2 or NL63 PVs, and Huh‐7 cells were seeded at 1 × 10^4^ cells per well in plates containing 229E PVs and CHO cells were seeded at 1 × 10^4^ cells per well in plates containing HKU1 PVs. Plates were incubated at 37°C and 5% CO_2_ for 48 h before lysis using Bright‐Glo and assaying luciferase reporter gene activity in relative light units (RLU) using a luminometer. All samples were repeated twice before calculating the final IC_50_. IC_50_ values were calculated for the neutralization assays based on 4‐parameter log‐logistic regression dose‐response curves. These curves were fit using Autoplate (Palmer et al, *under review*) and the R package drc.

### 2.6 Fluorescence assisted cell sorting (FACS) assay

HEK293T cells were transfected with an expression plasmid expressing wild‐type Spike glycoprotein of each of the four seasonal coronaviruses (HCoV‐NL63, HCoV‐229E, HCoV‐OC43, and HCoV‐HKU1). 48 h after transfection, cells were transferred into V‐bottom 96‐well plates (50 000 cells/well). Cells were incubated with sera (diluted at 1:50 in PBS) or anti‐human IgG Isotype negative control (Invitrogen 31154, diluted at 20 µg/ml in PBS) for 30 min, washed with FACS buffer (PBS, 1% FBS, 0.02% Tween 20) and stained with Goat anti‐human IgG (H + L) Alexa Fluor 647 Secondary Antibody (Invitrogen A‐21445, diluted at 20 µg/ml in FACS buffer), for 30 min in the dark. Cells were washed with FACS buffer and samples were run on a Attune NxT Flow Cytometer (Invitrogen) with a high‐throughput autosampler. Dead cells were excluded from the analysis by staining cells with 7‐Aminoactinomycin D (7‐AAD) and gating 7‐AAD negative live cells.

### 2.7 Statistical methods

#### 2.7.1 HCoV neutralization and COVID‐19 severity

We used multiple regression to compare HCoV neutralization titers between SARS‐CoV‐2 seropositive HCWs and COVID‐19 patients after accounting for differences in age and sex. Age and sex effects were reported after dropping nonsignificant HCW/patient terms. A linear model predicting HCoV neutralization was fit separately for NL63, HKU1, and 229E. All statistical analyses were performed using R.^25^

We fit a linear regression to predict COVID‐19 severity in 81 seropositive people. This model included the natural log of the SARS‐CoV‐2 pMN IC50 and a binary term indicating whether or not the sample came from a hospitalized COVID‐19 patient. We used an F ratio test to determine if the natural log of the NL63 pMN IC50 significantly improved the model fit. Plots of residuals, leverage, and qq‐plots were used to assess the assumptions of the model.

The WHO COVID‐19 severity score is ordinal so we also analyzed our data using a proportional odds logistic regression designed for ordinal variables to ensure our conclusions are robust to nonlinearity in the data. Similar to the linear regression, our proportional odds logistic regression predicted COVID‐19 severity in 81 seropositive people using the natural log of SARS‐CoV‐2 pMN IC50 and whether or not the sample came from a COVID‐19 patient as predictors. We used a likelihood ratio test to test if the natural log of NL63 pMN IC50 significantly predicted COVID‐19 severity after accounting for the other variables. The assumption of proportional odds was assessed by visualizing coefficients of logistic regression models predicting severity equal to, or greater than *i* for *i* equals 2‐7.

#### 2.7.2 Does SARS‐CoV‐2 exposure increase HCoV neutralization?

If SARS‐CoV‐2 infection increased HCoV antibody titer we would expect HCoV neutralization to be higher in seropositive samples. We compared HCoV neutralization between serostatus groups to test if SARS‐CoV‐2 infection increased HCoV neutralization. We used a linear model using serostatus, sex, and age as predictors.

To investigate the effect of vaccination against SARS‐CoV‐2 on NL63 neutralization we quantified NL63 neutralization of 21 HCW before and after receiving their first dose of the SARS‐CoV‐2 vaccination. The significance of any change before and after vaccination was calculated using a paired Wilcoxon signed‐rank test.

#### 2.7.3 Correlations between neutralization of different viruses and spike binding

We investigated the correlation between neutralization of SARS‐CoV‐2 and HCoVs using Spearman's rank. We also visualized all correlations between HCoV and SARS‐CoV‐2 neutralization and spike binding using a Spearman's rank correlation plot.^26^

## 3 RESULTS

### 3.1 Neutralizing antibodies against all three pseudotype HCoVs detected in plasma samples

**Figure 1:**
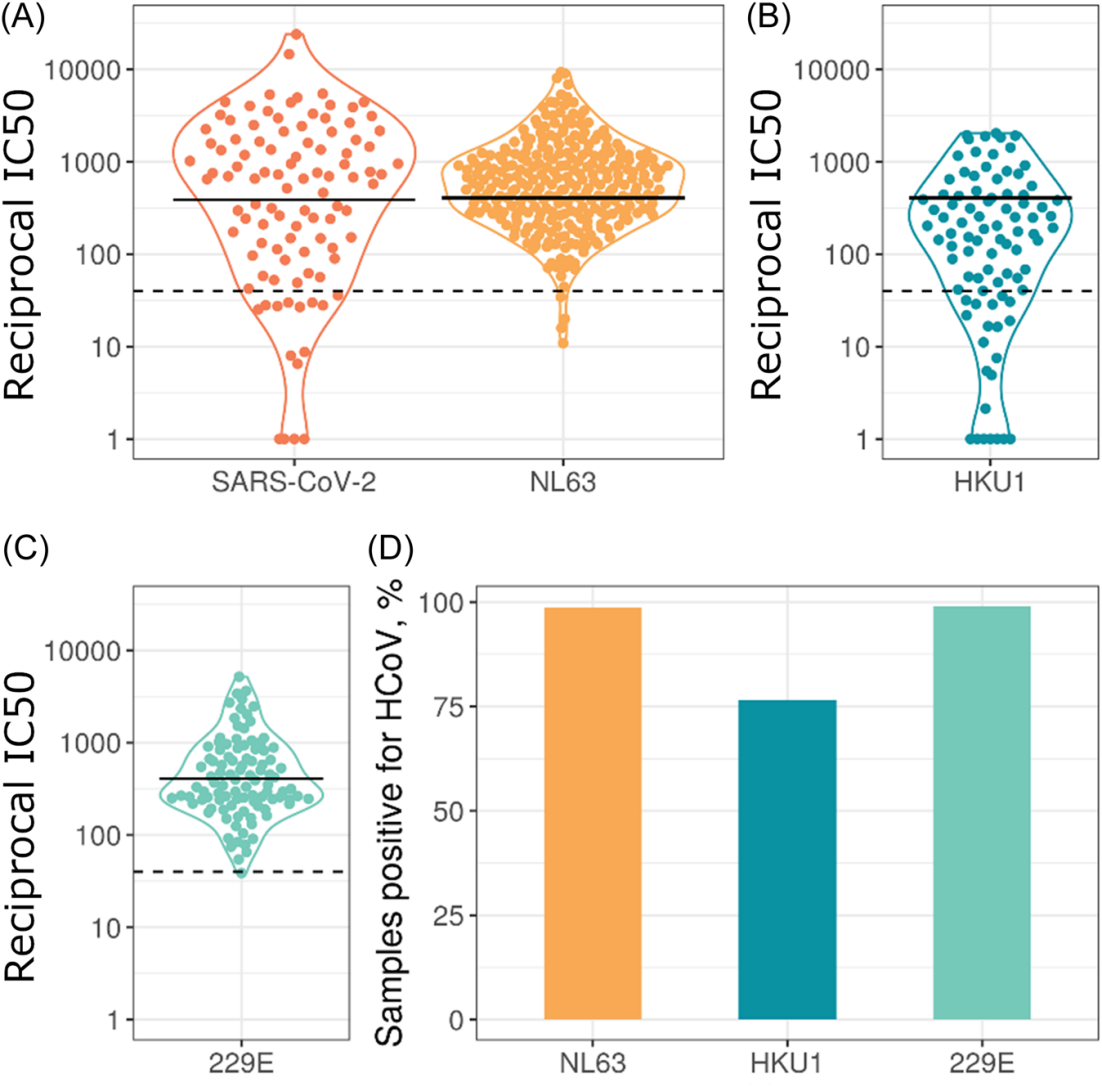
Neutralization IC_50_ values for HCoVs and SARS‐CoV‐2 (A–C). Solid lines represent geometric means, dashed horizontal lines indicate the cutoff chosen to define detectable HCoV neutralization. Due to the different cell lines used for HKU1 (B) and 229E (C), data points were plotted on separate graphs. 98.6% of 282 plasma samples neutralized NL63 (A), 76.4% of 89 samples neutralized HKU1 (B), and 99% of 100 samples neutralized 229E (C). The SARS‐CoV‐2 data only includes samples from seropositive individuals. Panel (D) shows the percentage of samples with detectable HCoV neutralization, IC_50_> 40. HCoVs, human coronaviruses; SARS‐CoV‐2, severe acute respiratory syndrome coronavirus 2

To assess if all samples (seronegative HCWs, seropositive HCW, and COVID‐19 patients) were positive for seasonal HCoVs we utilized pseudotype virus neutralization assays. Samples with neutralization IC_50_ over 40 were classed as neutralizing; the majority of samples neutralized all three HCoVs tested. We found that 98.6% of 282 plasma samples neutralized NL63, 76.4% of 89 samples neutralized HKU1, and 99% of 100 samples neutralized 229E (Figure 1). This illustrates the prevalence of HCoV infection and how common it is for people to have circulating neutralizing antibodies to HCoVs.

**Figure 2:**
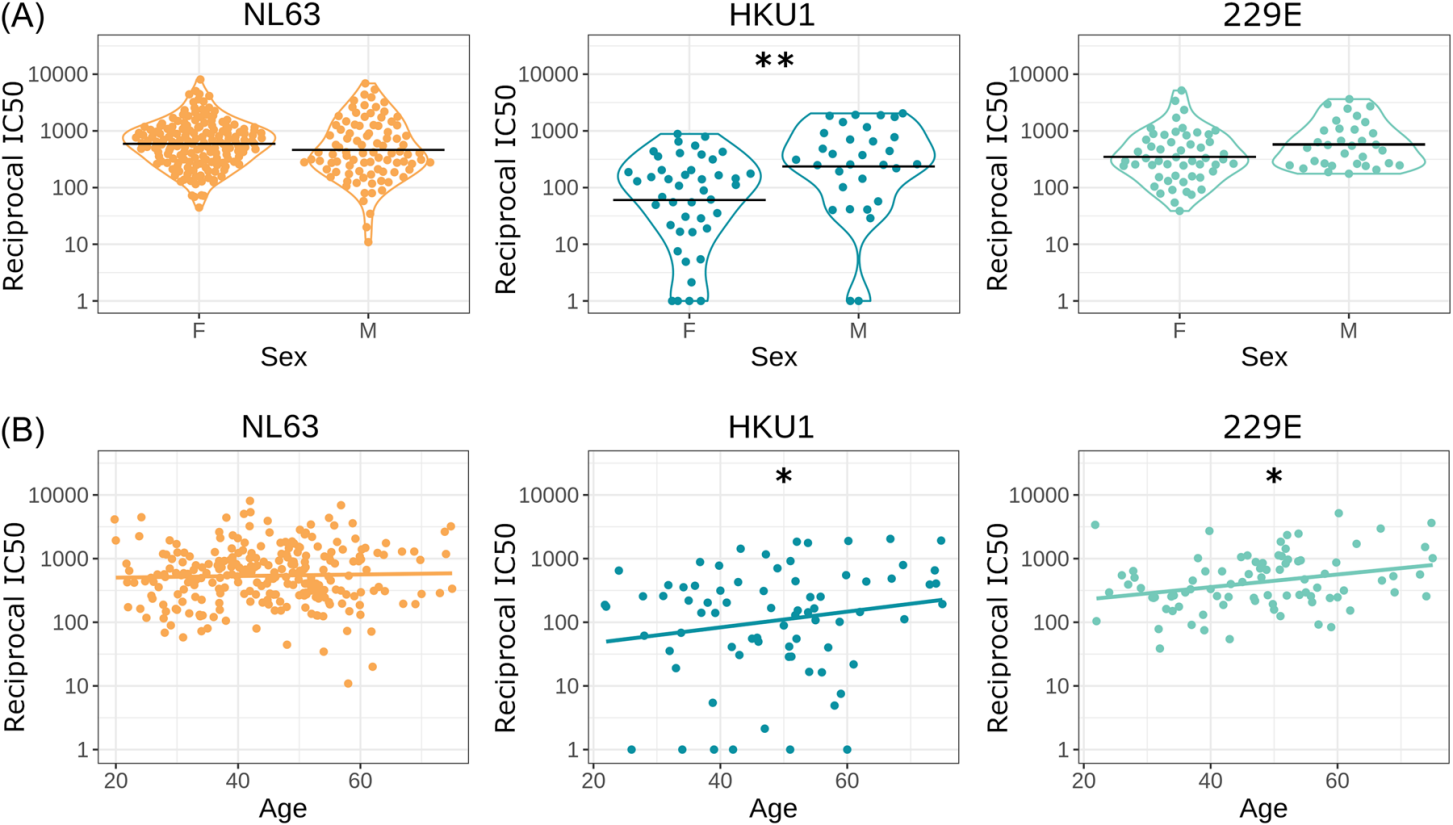
HCoV neutralization by demographic. Neutralizing IC_50_ value for HCoVs against sex (A) and age (B). Black horizontal lines represent geometric means, colored lines in are simple linear regression lines. Sample sizes: NL63 *n* = 84, HKU1 *n* = 43, 229E *n* = 47. We found a significant difference in HKU1 neutralization between sex (*p* = 0.004) and age (*p* = 0.039). We did not see any statistical significance in HCoVs NL63 nor 229E between sexes. We observed a significant difference in 229E neutralization and age (*p* = 0.037). *p < 0.05, **p < 0.01 ***p < 0.001. HCoVs, human coronaviruses

We found significant sex and age differences in neutralization titers for some HCoVs when analyzing SARS‐CoV‐2 seropositive samples (Figure 2). There was no significant effect for NL63 (sex *ß* = 0.06, SE = 0.23, *p* = 0.78; age *ß* = 0.01, SE = 0.01, *p* = 0.27, *n* = 84). For HKU1 there was a significant effect of sex and age (sex *ß* =1.9, SE = 0.62, *p* = 0.004; age *ß *= 0.05, SE = 0.02, *p* = 0.039, *n* = 43). For 229E there was a significant effect of age but not sex (sex *ß *= 0.47, SE = 0.26, *p* = 0.077, age *ß *= 0.02, SE = 0.01, *p* = 0.037, *n* = 47).

### 3.2 HCoV neutralization and COVID‐19 severity

**Figure 3:**
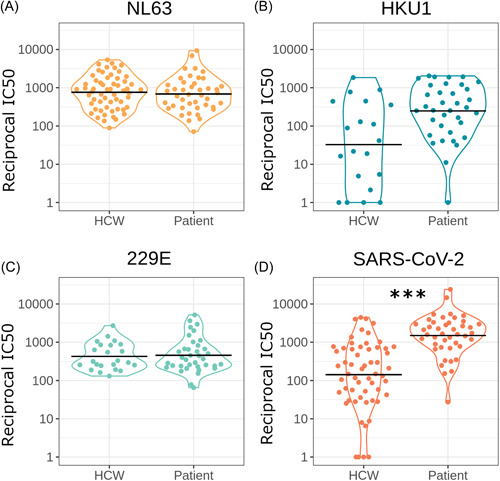
Comparing neutralization between seropositive HCWs and seropositive patients for NL63 (A) (*n* = 84) (*p* = 0.150), HKU1 (B) (*n* = 43) (*p* = 0.162), 229E (C) (*n* = 47) (*p* = 0.155), and SARS‐CoV‐2 (D) (*n* = 101) (*p* < 0.0001). Horizontal black lines indicate geometric means. *p < 0.05, **p < 0.01 ***p < 0.001. HCWs, healthcare workers; SARS‐CoV‐2, severe acute respiratory syndrome coronavirus 2

If HCoV immune responses modulate COVID‐19 severity we may observe a difference in HCoV neutralization between seropositive HCWs and COVID‐19 patients. However, after accounting for age and sex we found no significant difference in HCoV neutralization between SARS‐CoV‐2 seropositive HCWs and patients (*n* = 100) (NL63 *p* = 0.150 *n* = 84, HKU1 *p* = 0.162 *n* = 43, 229E *p* = 0.155 *n* = 47, Figure 3) (Table 1).

**Table 1:** Population characteristics of samples used in this study

Age	20–31	31–42	42–53	53–64	64–75		
	43	69	83	49	11		
**Sex**	**Female**	**Male**					
	163	92					
**Severity**	**1**	**2**	**3**	**4**	**5**	**6**	**7**
seronegative	109	59	13	0	0	0	0
seropositive	15	34	10	16	1	3	12

**Figure 4:**
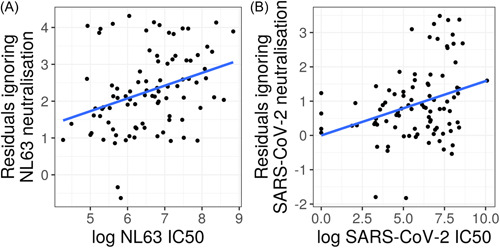
Partial residual plots for the natural log of NL63 (*n* = 91) (A) and SARS‐CoV‐2 (*n* = 91) (B) pMN IC_50_ values predicting COVID‐19 severity. These plots illustrate the effect of a variable on severity after accounting for other variables in the linear regression. This data suggests that NL63 neutralization is associated with COVID‐19 disease severity. COVID‐19, coronavirus disease‐2019; SARS‐CoV‐2, severe acute respiratory syndrome coronavirus 2

NL63 neutralization, the only HCoV to use the same ACE2 receptor for cell entry,^27^ was significantly associated with COVID‐19 severity. This positive relationship was found after accounting for SARS‐CoV‐2 neutralization and is illustrated in partial residual plots (Figure 4). This result was found in both our linear regression (*F* = 10.4, *df* = 1, *p* = 0.002, *n* = 91) (Table 2), and our proportional odds logistic regression (LR = 16.7, *df* = 1, *p* < 0.001, *n* = 91). This result seems to be driven by the difference between symptomatic and asymptomatic cases as the effect was only borderline significant when analyzing only symptomatic cases (LR = 3.5, *df* = 1, *p* = 0.06, *n* = 67). This suggests that NL63 neutralization is elevated in people who have suffered more than very mild COVID‐19.

**Table 2:** COVID‐19 severity linear regression coefficient estimates and standard errors

Coefficient	Estimate	Standard error	*p* Value
Intercept	−1.1	0.75	0.13
Ln SARS‐CoV‐2 pMN IC_50_	0.16	0.06	0.0088
Patient	2.9	0.26	<0.0001
Ln NL63 pMN IC_50_	0.35	0.11	0.0018

Abbreviations: COVID‐19, coronavirus disease‐2019; SARS‐CoV‐2, severe acute respiratory syndrome coronavirus 2.

### 3.3 Does SARS‐CoV‐2 exposure increase HCoV neutralization?

**Figure 5:**
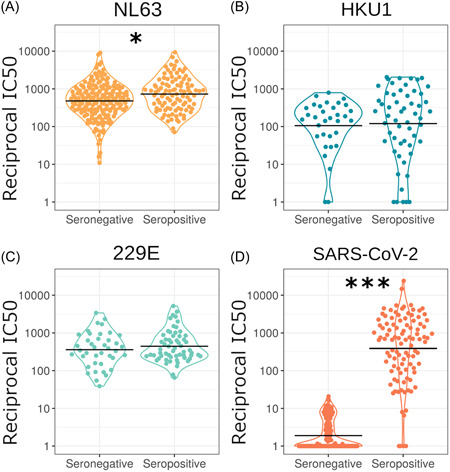
Comparing neutralization of SARS‐CoV‐2 seropositive and seronegative samples with the HCoVs (A–C). Our results revealed significant differences for NL63 (A) (*n* = 255) (*p* = 0.018) and SARS‐CoV‐2 (D) (*n* = 255) (p = <0.001). However, we observed no significance for HKU1 (B) (*n* = 75) (*p* = 0.143) and 229E (C) (*n* = 86) (*p* = 0.978). Horizontal black lines indicate geometric means. Samples were grouped as seropositive or seronegative regardless of being from patients or HCWs. Serostatus was based on IgG binding by Luminex assay or by SARS‐CoV‐2 pMN assay where a cut‐off reciprocal IC_50_ value was derived using pre‐pandemic sera as described in the methods or in Castillo‐Olivares et al.^19^ A small number of samples were classed as seropositive by IgG binding assay despite low neutralization. *p < 0.05, **p < 0.01 ***p < 0.001. HCWs, healthcare workers; SARS‐CoV‐2, severe acute respiratory syndrome coronavirus 2

To investigate if SARS‐CoV‐2 infection increases HCoV neutralization we compared SARS‐CoV‐2 seronegative and SARS‐CoV‐2 seropositive sample neutralization for NL63, HKU1, 229E, and for reference SARS‐CoV‐2 (Figure 5). We found a small but significant 1.5‐fold increase in geometric mean of NL63 neutralization after accounting for the effects of sex and age (serostatus *ß *= 0.43, SE = 0.14, *p* = 0.003; sex *ß* = −0.33, SE = 0.14, *p* = 0.018; age *ß *= 0, SE = 0.01, *p* = 0.90, *n* = 255). We found no difference in HCoV neutralization between seropositive and seronegative samples for HKU1 (serostatus *ß *= −0.73, SE = 0.50, *p* = 0.143; sex *ß* = 1.5, SE = 0.49, *p* = 0.002; age *ß *= 0.03, SE = 0.02, *p* = 0.150, *n* = 75) or 229E (serostatus *ß *= −0.01, SE = 0.24, *p* = 0.978; sex *ß *= 0.39, SE = 0.24, *p* = 0.104; age *ß *= 0.02, SE = 0.01, *p* = 0.021, *n* = 86). As expected, seropositive samples showed a large increase in SARS‐CoV‐2 neutralization (*n* = 255) (205‐fold increase, *p* < 0.001).

**Figure 6:**
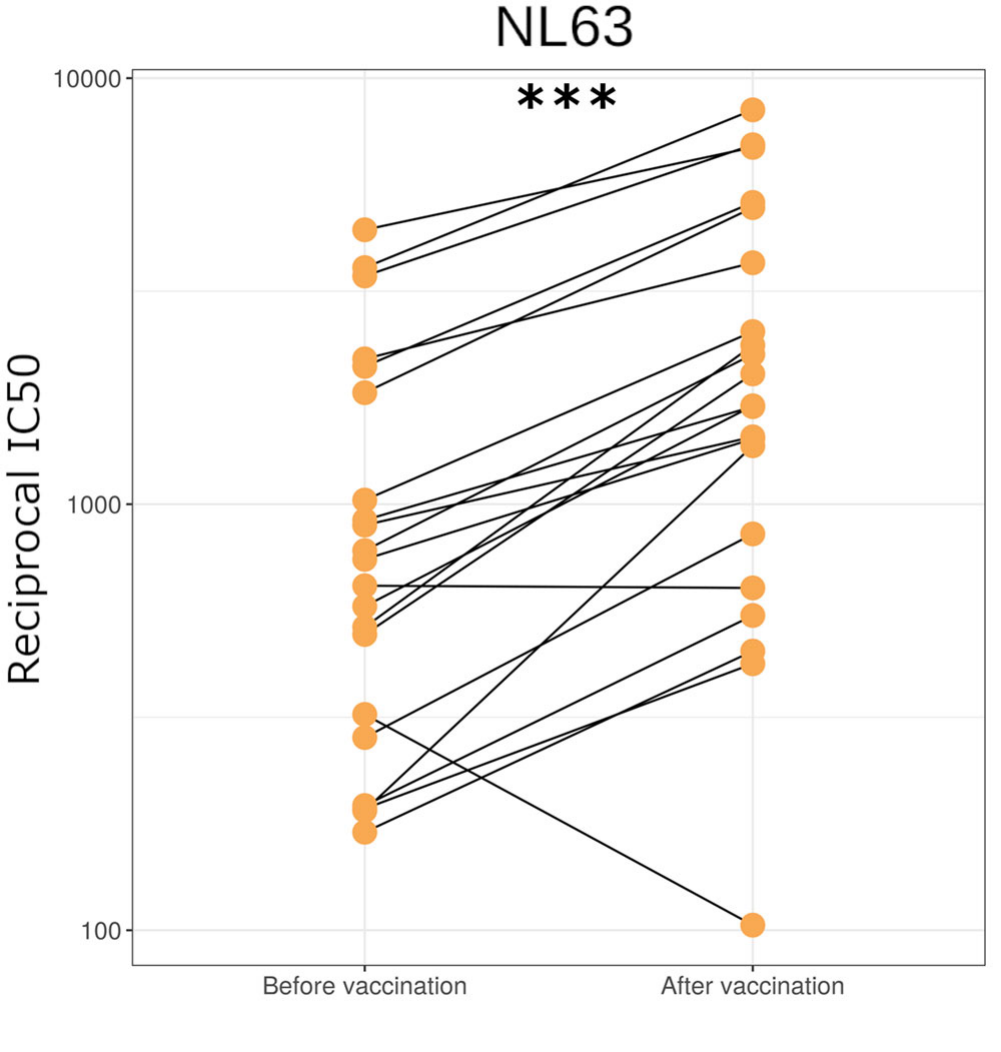
NL63 neutralization in HCWs before and approximately 1 month after first dose of SARS‐CoV‐2 vaccination (*n* = 21) (*p* = <0.001). *p < 0.05, **p < 0.01 ***p < 0.001. HCWs, healthcare workers; SARS‐CoV‐2, severe acute respiratory syndrome coronavirus 2

We also found that SARS‐CoV‐2 vaccination significantly increased NL63 neutralization (*p* = 0.0001, *n* = 21, Figure 6). We used paired pre‐ and postvaccination samples to identify a significant increase in NL63 neutralization postvaccination. The geometric mean of fold‐increase in NL63 neutralization, 2.2 was similar to the difference between seropositive and seronegative cases. Vaccination appears to cause a similar fold‐increase in NL63 neutralization to nearly all samples with the exception of two.

### 3.4 Correlations between neutralization of different viruses and spike binding

**Figure 7:**
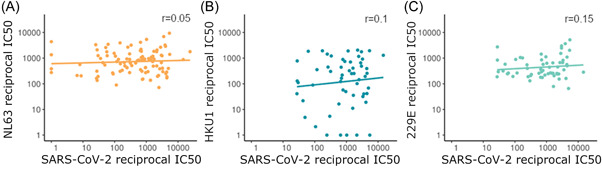
The relationship between SARS‐CoV‐2 neutralization and NL63 (*n* = 101) (A), HKU1 (*n* = 56) (B), and 229E (*n* = 60) (C) in SARS‐CoV‐2 seropositive samples with a linear line of best fit. Spearman's rank test reveals no significant correlation for each HCoV tested. HCoV, human coronaviruse; SARS‐CoV‐2, severe acute respiratory syndrome coronavirus 2

We found no correlation between HCoV neutralization and SARS‐CoV‐2 neutralization in SARS‐CoV‐2 seropositive individuals (Figure 7), suggesting that HCoV neutralizing antibodies do not neutralize SARS‐CoV‐2. The Spearman's rank correlation coefficient was nonsignificant for each HCoV tested, NL63 *r* = 0.05, *p* = 0.65, *n* = 101; HKU1 *r* = 0.1, *p* = 0.48, *n* = 56; 229E *r* = 0.15, *p* = 0.24, *n* = 60. This is in keeping with our finding that HCoV neutralization was not associated with lower COVID‐19 severity.

**Figure 8:**
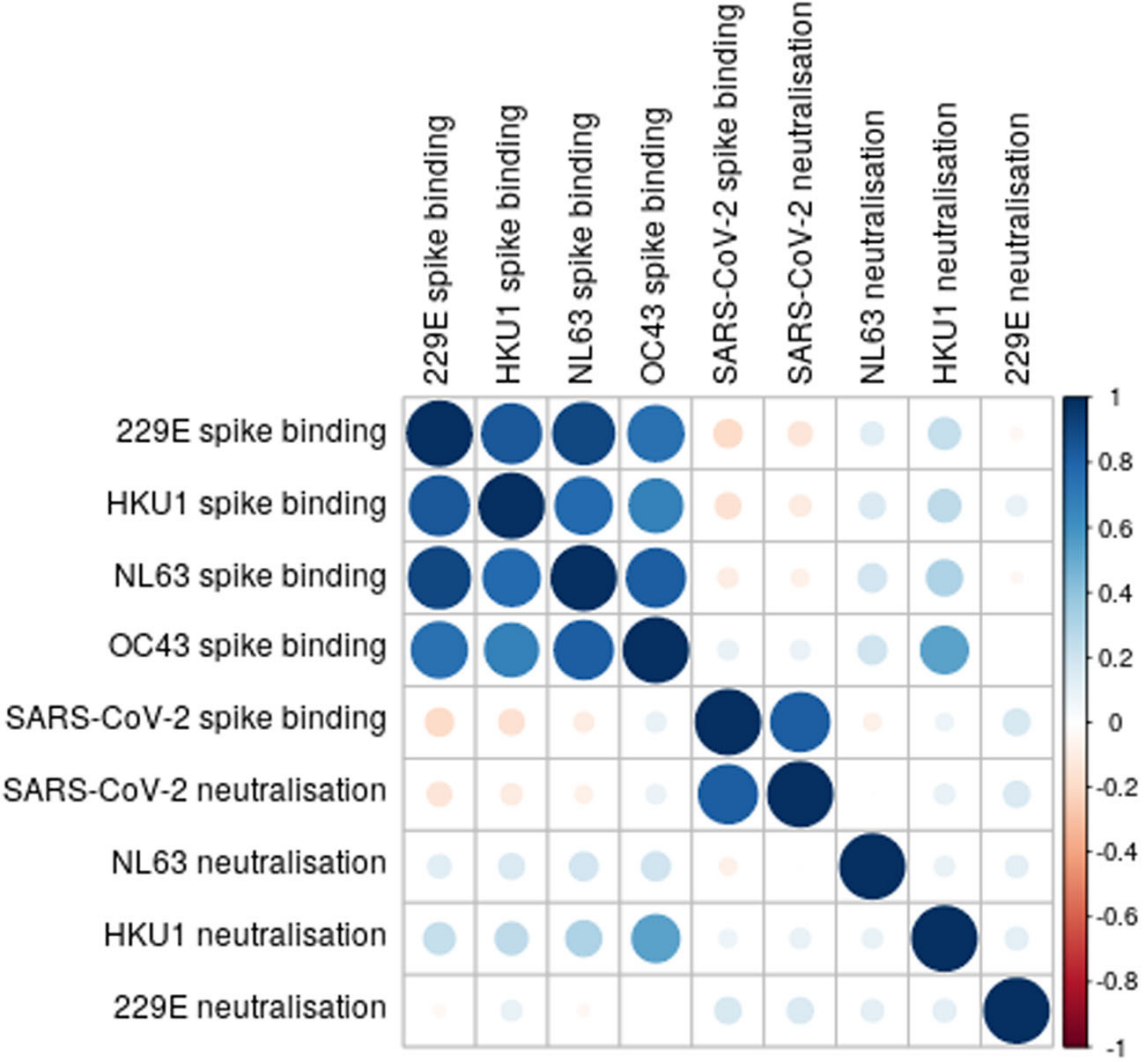
Correlation matrix of virus binding and neutralization. Larger darker circles indicate stronger correlations, as measured by Spearman's rank correlation coefficient. Blue circles indicate positive correlation and red circles indicate negative correlations. Sample sizes: SARS‐CoV‐2; *n* = 101. NL63; *n* = 101. HKU1; *n* = 56. 229E *n* = 60. SARS‐CoV‐2, severe acute respiratory syndrome coronavirus 2

Although SARS‐CoV‐2 spike binding was closely correlated with SARS‐CoV‐2 neutralization, there was little correlation between spike binding and neutralization for HCoVs. However, there was a strong positive correlation between spike binding to the different HCoVs (Figure 8).

## 4 DISCUSSION

HCoVs cause frequent mild infections in humans with most people becoming infected during infancy,^28^ and reinfections remain a common occurrence after approximately 12 months.^29^ Because of the frequency of HCoV infections much of the population will possess some immune response to one or more of the HCoVs. Therefore, it is important to identify any impact HCoV immune responses have on SARS‐CoV‐2, or the severity of COVID‐19 disease it causes. We found that almost all samples tested had antibodies that neutralized NL63 and 229E, and more than three‐quarters of all samples had neutralizing titers to HKU1. We found that HCoV neutralization was not associated with protection from COVID‐19. This result builds on previous work by Anderson et al.^14^ which showed that HCoV binding did not protect against COVID‐19. Despite similar conclusions, we found that HCoV spike binding did not correlate well with HCoV neutralization.

Given that NL63 was the HCoV most likely to influence COVID‐19 severity because it also targets the ACE2 receptor for cell entry, we analyzed a larger sample size for NL63 neutralization. Interestingly, we found that NL63 neutralization was positively correlated with COVID‐19 severity after accounting for SARS‐CoV‐2 neutralization using two separate statistical methods. This relationship is most clearly seen within HCW with mild disease, explaining why we did not find that patients had higher NL63 neutralization. A previous report found that all samples with severe COVID‐19 disease showed very low levels of NL63 neutralizing antibodies.^30^ However, this contrasts with our findings which demonstrate a range of NL63 neutralization titers in individuals with both high and low COVID‐19 severity scores.

Here, we present evidence that exposure to SARS‐CoV‐2 increases the neutralization of NL63 despite no cross‐neutralization. We show that SARS‐CoV‐2 vaccination increases neutralization of NL63 and that NL63 neutralization was higher in SARS‐CoV‐2 seropositive samples. One limitation of this study is the lack of paired samples immediately before and after infection to measure the effect of SARS‐CoV‐2 infection on NL63 neutralization directly. If moderate to severe COVID‐19 disease causes an increase in NL63 neutralization it would explain the observed relationship between COVID‐19 severity and NL63 neutralization. The increase in NL63 neutralization after vaccination suggests the possibility that SARS‐CoV‐2 vaccination may modestly boost immunity memory against NL63, rather than generate cross‐reactive antibodies since we observed very little correlation in cross‐reactivity. It remains to be seen whether vaccination against SARS‐CoV‐2 would result in increased protection against NL63.

Spike protein binding was highly correlated between the HCoVs; however, there was relatively little correlation between HCoVs and SARS‐CoV‐2. If the correlation between HCoV binding was driven by cross‐reactive antibodies we would also expect them to correlate with SARS‐CoV‐2 as it is more closely related to the betacoronavirus HCoVs than 229E and NL63 are. We interpret the lack of correlation in spike binding between SARS‐CoV‐2 as evidence that HCoV spike antibodies are likely not cross‐reactive but co‐occurring, that is, people who are exposed to one of the HCoVs are likely to be exposed to other HCoVs.^29^

We found that SARS‐CoV‐2 neutralization does not correlate with neutralization of any HCoV we tested. At first, this seems to contradict several studies reporting cross‐reactive binding and neutralization^7^, ^8^; however, these studies found only a very small proportion of people not exposed to SARS‐CoV‐2 displayed cross‐reactive antibodies. Ng et al.^7^ found that less than 1% of pre‐pandemic samples showed SARS‐CoV‐2 RBD binding antibodies. This suggests that the majority of HCoV antibodies do not cross‐react with SARS‐CoV‐2 and is in keeping with our results that HCoV neutralization is not correlated with SARS‐CoV‐2 neutralization, nor does it provide protection against COVID‐19, which is consistent with a similar study.^31^ On the other hand, a study observed that a recent HCoV infection may provide some degree of protection.^32^ We do not have information on timing of HCoV infection so cannot test this relationship in this study.

One of the limitations of pseudotype viruses is that they possess only the spike protein; therefore, the effects of other viral proteins remain in question. The nucleocapsid protein (N) shows highly conserved motifs in the N‐terminus, observed across a wide range of the HCoVs and SARS‐CoV‐2.^33^ Cross‐reactivity between SARS‐CoV‐1 N‐antibodies and several animal coronaviruses were previously described, despite lack of cross‐reactive spike antibodies.^34^ Similarly, several reports have found cross‐reactive antibodies between HCoVs and SARS‐CoV‐2 S, M, and N proteins.^7^, ^8^, ^9^ Importantly, a report observed that N‐antibodies of several viruses activate the TRIM21 pathway, which then drives cytotoxic T‐cell activation.^35^ This highlights the multifaceted defence mechanisms against SARS‐CoV‐2 and the HCoVs, of which further studies in each of these areas may contribute to the debate regarding cross‐protection between SARS‐CoV‐2 and HCoVs.

## AUTHOR CONTRIBUTIONS

The study was conceptualized by David A. Wells, Diego Cantoni, Javier Castillo‐Olivares, Nigel Temperton, and Jonathan L. Heeney. Hospital patient enrollments and sample collections were coordinated by Gianmarco Raddi and Helen Baxendale. Processing of blood samples was done by Angalee Nadesalingam, Andrew Chan, and Peter Smith. Neutralization assays were carried out by Diego Cantoni, Martin Mayora‐Neto, Cecilia Di Genova, George Carnell, and Alexander Sampson FACS assay was carried out by Matteo Ferrari. Data processing and statistical analyses were done by David A. Wells. All authors provided critical feedback on project direction, data analysis, and manuscript drafts. All authors had access to the data that was generated in this study. All authors approved the submitted version.

## CONFLICT OF INTEREST

David A. Wells was employed to DIOSynVax at the time of this study. Matteo Ferrari, and Jonathan Heeney are currently employed/affiliated to DIOSynVax company. The authors declare that the research was conducted in the absence of any commercial or financial relationships that could be construed as a potential conflict of interest. DIOSynVax did not provide any funding toward this study.

## Data Availability

The data that support the findings of this study are available from the corresponding author upon reasonable request. All raw data used in this manuscript is available upon reasonable request to the corresponding authors.
